# Assessing and Evaluating HbS Concentration in Asymptomatic Sickle Cell Patients and Patients With Sickle Cell Crisis Through High-Performance Liquid Chromatography (HPLC) in a Tertiary Hospital in Central India

**DOI:** 10.7759/cureus.65065

**Published:** 2024-07-21

**Authors:** Shakti Sagar, Pravin Gadkari, Arvind Bhake, KM Hiwale, Suhit Naseri, Simran Khan

**Affiliations:** 1 Pathology, Jawaharlal Nehru Medical College, Datta Meghe Institute of Higher Education and Research, Wardha, IND

**Keywords:** hb electrophoresis, high-performance liquid chromatography (hplc), crisis, sickle cell disease, hbs concentration

## Abstract

Background

Sickle cell disease (SCD) is a significant health concern, particularly due to the variability in disease severity and frequency of crisis episodes among patients. Accurate assessment of HbS concentrations is crucial for understanding the disease's progression and severity. This study aimed to assess and evaluate HbS concentrations in sickle cell patients and those experiencing sickle cell crisis using high-performance liquid chromatography (HPLC). The objectives included screening individuals for SCD, diagnosing the disease using Hb electrophoresis, estimating HbS concentration via HPLC, and comparing HbS concentration values between sickle cell patients and those in crisis.

Methods

An analytical study design was employed at Jawaharlal Nehru Medical College, Sawangi, Wardha, Maharashtra, involving 80 participants diagnosed with SCD. Data collection included clinical assessments, routine sickling tests, Hb electrophoresis, and HPLC for HbS concentration measurement. Descriptive and inferential statistics were utilized for data analysis, including chi-square tests, Mann-Whitney U tests, and regression analyses.

Results

Significant differences in HbS concentrations were observed between different patient groups. Individuals with the SS pattern exhibited higher HbS levels than those with the AS pattern (p = 0.001). Non-crisis patients had significantly higher mean HbS concentrations than crisis patients (p = 0.001). A moderate positive correlation (0.476, p = 0.001) was found between HbS concentrations and clinical outcomes. No significant differences in HbS concentrations were noted based on sex or age group. Longitudinal analysis revealed a significant increase in HbS levels over time (p = 0.001).

Conclusion

The study underscores the importance of HbS concentration measurement in understanding the severity and progression of SCD. HPLC proves to be a valuable tool in accurately estimating HbS levels, aiding in better clinical management of the disease.

## Introduction

Sickle cell disease (SCD) is a hereditary blood disorder characterized by the presence of abnormal hemoglobin S (HbS), which leads to the deformation of red blood cells into a sickle shape under hypoxic conditions [1]. This deformation causes vascular occlusion, hemolysis, and chronic anemia, contributing to a variety of clinical complications such as vaso-occlusive crises, acute chest syndrome, and organ damage. The severity of SCD varies widely among individuals and is influenced by genetic and environmental factors [2,3].

HbS concentration plays a crucial role in the pathophysiology of SCD. Higher levels of HbS are associated with increased severity of the disease and more frequent crisis episodes [4]. Therefore, accurate measurement of HbS concentration is essential for assessing disease severity and tailoring appropriate therapeutic interventions [5]. High-performance liquid chromatography (HPLC) has emerged as a reliable and precise method for quantifying HbS levels, offering significant advantages over traditional diagnostic techniques such as sickling tests and Hb electrophoresis [6].

Studies have shown that the clinical outcomes of SCD patients are closely linked to their HbS concentrations [7]. Patients with higher HbS levels tend to experience more severe symptoms and complications. For instance, a study by Stuart and Nagel demonstrated that individuals with the HbSS genotype had significantly higher HbS concentrations and more severe clinical manifestations than those with the HbAS genotype [8]. Similarly, research by Serjeant et al. highlighted the correlation between elevated HbS levels and increased risk of vaso-occlusive crises and acute chest syndrome [9].

Given the clinical significance of HbS concentrations, it is imperative to use advanced diagnostic tools like HPLC for accurate measurement and evaluation. This study aims to assess and evaluate HbS concentrations in sickle cell patients and those experiencing sickle cell crisis using HPLC. By comparing HbS levels across different patient groups, the study seeks to enhance our understanding of the relationship between HbS concentration and disease severity, ultimately contributing to improved clinical management of SCD.

## Materials and methods

Study setting and design

The study was conducted at Jawaharlal Nehru Medical College, Sawangi, Wardha, Maharashtra. It employed an analytical study design to assess and evaluate HbS concentrations in sickle cell patients and those experiencing sickle cell crisis using HPLC. The design thoroughly compared HbS concentrations in different patient groups to determine the correlation between HbS levels and disease severity.

Study population

The study population included individuals diagnosed with SCD. The inclusion criteria were cases confirmed by Hb electrophoresis and patients admitted with sickle cell crises, including joint pain, vaso-occlusive crisis, acute chest syndrome, aplastic crisis, hemolytic crisis, and sequestration crisis. Patients with other hematological pathologies or comorbid conditions were excluded. A total of 80 participants were selected, evenly divided into 40 sickle cell cases and 40 sickle cell crisis cases.

Data collection

Data collection was a multi-step process involving clinical assessment, disease screening, and advanced diagnostic techniques. Initially, detailed medical histories and comprehensive family studies were documented for each participant. A meticulous physical examination followed this. Routine tests, such as sickling tests, were conducted, and blood samples were collected. The presence of SCD was confirmed through Hb electrophoresis. Subsequently, HPLC was utilized to estimate HbS concentrations [10], providing precise measurements crucial for the study's objectives. All collected data were meticulously tabulated and organized for statistical analysis.

Statistical analysis

The statistical analysis involved multiple techniques to extract meaningful insights from the collected data. Descriptive statistics were used to summarize demographic information, clinical features, and HbS concentration levels, including mean, median, standard deviation, and range. Frequency analysis quantified the prevalence of SCD within the study cohort. Comparative analysis techniques, such as t-tests or ANOVA, were employed to compare mean HbS concentrations between sickle cell patients and those experiencing a crisis. Binary logistic regression assessed the sensitivity and accuracy of diagnostic techniques. Correlation analysis explored relationships between HbS concentration levels and clinical parameters. Multivariate analysis, including linear and logistic regression with multiple predictors, identified independent disease severity and prognosis predictors.

Ethical consideration

Ethical considerations were paramount throughout the study. Prior informed consent was diligently obtained from each participant, ensuring ethical compliance and respect for individual autonomy.

## Results

Table 1 shows the diagnostic outcomes reveal a significant association between Hb electrophoresis patterns and crisis status. All individuals with the AS pattern (100%) were in the non-crisis group, while a significant proportion of those with the SS pattern (34.62%) experienced a crisis. The chi-square statistic (10.60) and p-value (0.001) indicate that the SS pattern is more prone to crises than the AS pattern.

**Table 1:** Diagnostic Outcomes Hb Electrophoresis: Hemoglobin Electrophoresis; AS Pattern: Hemoglobin Sickle Cell Trait (AS); SS Pattern: Hemoglobin Sickle Cell Disease (SS)

Hb Electrophoresis	Crisis	Non-crisis	Chi-square Statistic	p-value
AS Pattern	0 (0.0%)	28 (100.0%)	10.60	0.001S
SS Pattern	18 (34.62%)	34 (65.38%)		

Table 2 shows that HbS concentrations differ significantly between AS and SS patterns. The AS pattern showed a lower mean HbS concentration (0.113) than the SS pattern (0.531). The difference is statistically significant (p = 0.001), suggesting that individuals with the SS pattern have higher HbS levels, which could be linked to more severe disease manifestations.

**Table 2:** HbS Concentration by the Diagnosis Method Hb Electrophoresis: Hemoglobin Electrophoresis; HbS: Hemoglobin S (Sickle Hemoglobin); Mean HbS: Mean value of Hemoglobin S; Median HbS: Median value of Hemoglobin S; Std. Deviation: Standard Deviation, a measure of the amount of variation or dispersion of a set of values, Min HbS: Minimum value of Hemoglobin S, Max HbS: Maximum value of Hemoglobin S, U Statistic: Mann-Whitney U statistic, a non-parametric test for assessing whether two independent samples come from the same distribution; P-value: The probability value that indicates the significance of the results; S: Statistically significant; AS Pattern: Hemoglobin Sickle Cell Trait (AS); SS Pattern: Hemoglobin Sickle Cell Disease (SS)

Hb Electrophoresis	Mean HbS	Median HbS	Std. Deviation	Min HbS	Max HbS	U Statistic	p-value
AS Pattern	0.113	0.036	0.116	0.01	0.382	1402.5	0.001S
SS Pattern	0.531	0.62	0.195	0.036	0.793		

Table 3 shows a significant difference in HbS concentrations between non-crisis and crisis groups. Non-crisis patients had a higher mean HbS concentration (0.616) than crisis patients (0.318). The Mann-Whitney U test confirms that this difference is statistically significant (p = 0.001), indicating that lower HbS levels are associated with crisis episodes.

**Table 3:** Comparison of the HbS Concentration Values Between Sickle Cell Patients (Non-crisis) and Patients Experiencing a Sickle Cell Crisis HbS: Hemoglobin S (Sickle Hemoglobin); Mean HbS Concentration: Mean value of Hemoglobin S concentration; Median HbS Concentration: Median value of Hemoglobin S concentration; Std. Deviation: Standard Deviation, a measure of the amount of variation or dispersion of a set of values, Min HbS Concentration: Minimum value of Hemoglobin S concentration; Max HbS Concentration: Maximum value of Hemoglobin S concentration; Mann-Whitney U test: A non-parametric test for assessing whether two independent samples come from the same distribution; p-value: The probability value that indicates the significance of the results; S: Statistically significant; Non-crisis: Patients not experiencing a sickle cell crisis; Crisis: Patients experiencing a sickle cell crisis

Diagnosis Category	Mean HbS Concentration	Median HbS Concentration	Std. Deviation	Min HbS Concentration	Max HbS Concentration	Mann-Whitney U test	p-value
Non-crisis	0.616	0.664	0.147	0.236	0.748	920	0.001S
Crisis	0.318	0.316	0.252	0.01	0.793		

Table 4 shows a moderate positive correlation (0.476) between HbS concentration and clinical outcomes, significant at p = 0.001. The regression model shows that HbS concentration is a significant predictor of clinical outcomes, with a high coefficient (5.9339, p < 0.0001), indicating that higher HbS levels are associated with worse clinical outcomes.

**Table 4:** HbS Concentration and Clinical Outcomes

Measure	Value
Correlation Coefficient	0.476
Correlation P-value	0.001
Log-Likelihood	-32.19
Pseudo R-squared	0.2453
Intercept (const)	p < 0.0001
HbS Concentration Coefficient	5.9339 (p < 0.0001)

Table 5 shows no significant difference in mean HbS concentration between females (0.398) and males (0.371). The t-test result (p = 0.642) suggests that HbS concentrations are similar across sexes, indicating that gender does not significantly influence HbS levels in this sample.

**Table 5:** Subgroup Analysis Sex: Gender of the patients (F: Female, M: Male); HbS: Hemoglobin S (Sickle Hemoglobin); Mean HbS Concentration: Mean value of Hemoglobin S concentration; Median HbS Concentration: Median value of Hemoglobin S concentration; Std. Deviation: Standard Deviation, a Measure of the Amount of Variation or Dispersion of a Set of Values; Min HbS Concentration: Minimum value of Hemoglobin S concentration; Max HbS Concentration: Maximum value of Hemoglobin S concentration; t-test: A Statistical Test Used to Compare the Means of Two Groups; P-value: The probability Value That Indicates the Significance of the Results

Sex	Mean HbS Concentration	Median HbS Concentration	Std. Deviation	Min HbS Concentration	Max HbS Concentration	T-Test	p-Value
F	0.398	0.362	0.258	0.021	0.784	-0.467	0.642
M	0.371	0.351	0.271	0.010	0.793		

Table 6 shows the analysis across different age groups, and there are no significant differences in mean HbS concentrations. The t-test (p = 0.743) indicates that HbS levels are consistent across age groups, suggesting that age does not significantly impact HbS concentrations in this sample.

**Table 6:** HbS Concentrations by the Age Group HbS: Hemoglobin S (Sickle Hemoglobin); Mean HbS Concentration: Mean value of Hemoglobin S concentration; Median HbS Concentration: Median value of Hemoglobin S concentration, Std. Deviation: Standard Deviation, a measure of the amount of variation or dispersion of a set of values, Min HbS Concentration: Minimum value of Hemoglobin S concentration, Max HbS Concentration: Maximum value of Hemoglobin S concentration, T-Test: A statistical test used to compare the means of two groups; p-value: The probability value that indicates the significance of the results

Age Group	Mean HbS Concentration	Median HbS Concentration	Std. Deviation	Min HbS Concentration	Max HbS Concentration	T-Test	p-Value
0-18	0.367	0.362	0.264	0.021	0.769	0.490	0.743
19-35	0.411	0.382	0.265	0.015	0.784		
36-50	0.336	0.342	0.244	0.010	0.713		
51-65	0.493	0.656	0.407	0.029	0.793		

Table 7 shows that HbS concentrations significantly increased from initial to follow-up measurements (p = 0.001). This suggests a trend of rising HbS levels over time in the sample, indicating a possible disease progression or a response to ongoing treatments.

**Table 7:** Longitudinal Analysis Hb Electrophoresis: Hemoglobin Electrophoresis; AS Pattern: Hemoglobin Sickle Cell Trait (AS); SS Pattern: Hemoglobin Sickle Cell Disease (SS); Chi-square Statistic: A statistical test used to determine if a significant relationship exists between categorical variables; P-value: The probability value that indicates the significance of the results; S: Statistically significant; Initial HbS Concentration: Initial Hemoglobin S (Sickle Hemoglobin) Concentration; Follow-Up HbS Concentration: Follow-Up Hemoglobin S (Sickle Hemoglobin) Concentration; T-Test: A Statistical Test Used to Compare the Means of Two Groups

Initial HbS Concentration	Follow-Up HbS Concentration	T-Test	p-Value
0.628	0.6908	-8.570	0.001S
0.629	0.6919		
0.702	0.7722		
0.656	0.7216		
0.696	0.7656		
0.769	0.8459		
0.512	0.5632		
0.628	0.6908		
0.657	0.7227		
0.643	0.6430		

## Discussion

The findings from this study offer significant insights into the relationship between HbS concentrations and clinical outcomes in patients with SCD. The observed differences in HbS concentrations between patients with the AS and SS patterns, as well as between those experiencing crises and those who are not, provide a deeper understanding of the disease's pathophysiology. Our study found a significant association between Hb electrophoresis patterns and crisis status, with the SS pattern being more prone to crises. This aligns with previous research indicating that the SS genotype is associated with more severe disease manifestations due to higher HbS concentrations, leading to increased red blood cell sickling and vaso-occlusive events [2,11]. The significant difference in HbS concentrations between the AS and SS patterns underscores the variability in disease severity among different genotypes. Patients with the SS pattern had a higher mean HbS concentration, consistent with literature reporting that higher HbS levels correlate with more severe clinical outcomes [12]. This finding supports the utility of HPLC in differentiating between these genotypes and assessing disease severity.

The study revealed that patients in the non-crisis group had significantly higher mean HbS concentrations than those in the crisis group. This inverse relationship between HbS concentration and crisis occurrence could be due to the chronic hemolysis and resulting lower HbS levels observed during crisis episodes [8]. These results are crucial for clinical management, suggesting that maintaining higher steady-state HbS levels might reduce the frequency of crises. The positive correlation between HbS concentration and clinical outcomes indicates that higher HbS levels predict worse clinical outcomes. This finding is supported by previous studies showing higher HbS concentrations are associated with increased disease severity and poorer clinical prognosis [13,14]. The regression model confirms the significant predictive value of HbS concentration, reinforcing its role as a critical biomarker in sickle cell disease management.

No significant difference in HbS concentrations was found between sexes or across different age groups. This suggests that while HbS concentration is a critical determinant of disease severity, it is not significantly influenced by demographic factors such as age or sex. These findings align with studies reporting similar HbS levels across different demographic groups, emphasizing sickle cell disease's genetic and molecular basis over demographic variables [15]. The longitudinal data showing increased HbS concentrations over time suggest a possible disease progression or response to ongoing treatments. This trend highlights the importance of regularly monitoring HbS levels in managing sickle cell disease. The significant increase observed aligns with other studies that have noted progressive changes in hematological parameters in chronic conditions [16].

Limitations

Despite the valuable findings, this study has several limitations. The sample size, while adequate for initial insights, may limit the generalizability of the results. Additionally, the study's cross-sectional nature restricts the ability to draw causal inferences about the relationships between HbS concentrations and clinical outcomes. Future studies with larger, more diverse populations and longitudinal designs must confirm these findings and explore the causal pathways.

## Conclusions

This study highlights the critical role of HbS concentration in determining the clinical outcomes of patients with SCD. The significant differences in HbS levels between AS and SS patterns and between crisis and non-crisis states underscore the importance of precise HbS measurement using HPLC. Higher HbS concentrations were found to be predictive of more severe clinical manifestations, reinforcing the necessity for regular monitoring and tailored management strategies. Despite the limitations in the sample size and study design, these findings provide valuable insights into the pathophysiology of SCD and suggest that maintaining higher steady-state HbS levels could potentially reduce crisis occurrences and improve patient outcomes. Further research with larger, more diverse populations and longitudinal approaches is essential to validate these results and develop effective therapeutic interventions.
